# Alteration of Golgi Structure by Stress: A Link to Neurodegeneration?

**DOI:** 10.3389/fnins.2015.00435

**Published:** 2015-11-12

**Authors:** Eduardo A. Alvarez-Miranda, Markus Sinnl, Hesso Farhan

**Affiliations:** ^1^Department of Industrial Engineering, Universidad de TalcaCuricó, Chile; ^2^Department of Statistics and Operations Research, University of ViennaVienna, Austria; ^3^Biotechnology Institute ThurgauKreuzlingen, Switzerland; ^4^Department of Biology, University of KonstanzKonstanz, Germany

**Keywords:** Golgi, Alzheimers disease, neurodegenration, computational modeling, protein–protein interactions, cellular stress response

## Abstract

The Golgi apparatus is well-known for its role as a sorting station in the secretory pathway as well as for its role in regulating post-translational protein modification. Another role for the Golgi is the regulation of cellular signaling by spatially regulating kinases, phosphatases, and GTPases. All these roles make it clear that the Golgi is a central regulator of cellular homeostasis. The response to stress and the initiation of adaptive responses to cope with it are fundamental abilities of all living cells. It was shown previously that the Golgi undergoes structural rearrangements under various stress conditions such as oxidative or osmotic stress. Neurodegenerative diseases are also frequently associated with alterations of Golgi morphology and many stress factors have been described to play an etiopathological role in neurodegeneration. It is however unclear whether the stress-Golgi connection plays a role in neurodegenerative diseases. Using a combination of bioinformatics modeling and literature mining, we will investigate evidence for such a tripartite link and we ask whether stress-induced Golgi arrangements are cause or consequence in neurodegeneration.

## Introduction

The Golgi apparatus is an organelle that is best known for its roles in post-translational protein modification and in secretory trafficking. In addition, the Golgi is increasingly being viewed as a signaling hub, which is not only able to respond to environmental factors, but it also able to modulate the outcome of signaling cascades by housing signaling molecules (Farhan and Rabouille, 2011; Cancino and Luini, 2013). As will be discussed later in greater detail, one of the signals that the Golgi is responsive to is cellular stress that was shown to induce prominent alterations of Golgi morphology.

Cells across all organisms have evolved adaptive mechanisms to survive adverse environmental conditions such as limited availability of nutrients, too high or low temperatures, non-physiologic pH, changes in tonicity, exposure to oxidative radicals, or the accumulation of toxic protein species. How cells deal with these stressors determines the fate of the cell, which might be survival, death, or malignant transformation. Therefore, it is not surprising that cellular stress responses were suggested to be involved in a plethora of diseases among them neurodegenerative disorders (Bhat et al., 2015; Coppedè and Migliore, 2015), which are the focus of the current work. Most diseases from that spectrum feature structural and functional alterations of the Golgi apparatus. Because stress signaling also affects the Golgi, we are tempted to speculate that the stress-Golgi connection is relevant for neurodegeneration. However, whether this is the case remains unclear. What has also not been investigated so far is the extent of the connections between stress and Golgi regulators. Another important question in this context is whether the Golgi simply acts as a receiver of stress signals, or whether we can infer evidence for the Golgi sending signals that modulate, stress, neurodegeneration, or both.

The goal of the current work is to use a computational biology approach to build a network of protein–protein interactions (PPI) to identify the extent of connections between stress signaling pathways and putative Golgi regulators. To do so, we will make use of the results of different RNAi screens that uncovered numerous potential regulators of Golgi morphology. The inferred network can be used to search for signatures of relevance for neurodegeneration. We will first review and introduce the relevant literature on the Golgi apparatus in neurodegenerative diseases and then the evidence for stress signaling and its connection to this organelle. We will also briefly introduce the different RNAi screens that were carried out in the past to investigate the secretory pathway and discuss the rationale behind inclusion and exclusion of their hits.

## The golgi in neurodegenerative diseases

A cellular event that has been observed in several neurodegenerative diseases is the fragmentation of the Golgi apparatus (Fan et al., 2008). In a mouse model of amyotrophic lateral sclerosis (ALS), severe Golgi fragmentation in motoneurons was observed, which was found to be due to loss of the Golgi-localized tubulin-binding cofactor E (TBCE; Bellouze et al., 2014). Depletion of TBCE not only resulted in a defect of microtubules biogenesis at the Golgi, but also affected vesicular transport from the Golgi to the endoplasmic reticulum (ER). This paper provides a potential mechanistic link between functional and structural alteration of the Golgi and a neurodegenerative disorder. The link between Golgi fragmentation and ALS was also reported in other studies. A transgenic mouse ALS model expressing mutated superoxide dismutase 1 (SOD1) displayed an up-regulation of the microtubule-depolymerizing protein stathmin (Strey et al., 2004) and a similar dysregulation of vesicular transport. In addition, Golgi fragmentation was also described as a result of inhibition of ER-to-Golgi trafficking (Cutrona et al., 2013). In Alzheimer's Disease (AD), Golgi fragmentation is linked to the serine/threonine kinase CDK5 shown to phosphorylate Golgi matrix proteins such as GM130 (Sun et al., 2008) and GRASP65 (Joshi et al., 2014). Tau, a microtubule binding protein that forms aggregates in AD was also shown to be phosphorylated by CDK5, an event thought to contribute to formation of neurofibrillary tangles (Castro-Alvarez et al., 2014) as well as leading to Golgi fragmentation (Liazoghli et al., 2005). Phosphorylation of tau subsequent to Golgi fragmentation has also been reported (Jiang et al., 2014). However, while the case of CDK5 in AD is reasonably well understood, we lack a broad and systematic understanding of the signaling pathways that regulate Golgi architecture under conditions relevant to neurodegeneration.

## The golgi under stress

All organelles of the secretory pathway receive signaling inputs initiated by external stimuli. Thereby, these organelles are capable of sensing the presence or absence of growth factors, nutrients, and amino acids (Farhan et al., 2010; Farhan and Rabouille, 2011; Zacharogianni et al., 2011). Among the signals that the Golgi responds to is cellular stress. Several stresses have been already reported to affect this organelle and alterations induced by osmotic stress were among the first to be reported. Because the plasma membrane is freely permeable to water, cells have evolved evolutionary highly conserved mechanisms to cope with changes in the osmolarity of their surrounding medium. In mammalian cells, hypertonic treatment was reported more than two decades ago to inhibit ER-to-Golgi transport (Docherty and Snider, 1991). Hypotonic stress induces tubulation of Golgi membranes (Lee and Linstedt, 1999), whereas hyperosmotic conditions induce its fragmentation and redistribution of the Golgi back to the ER (Lee and Linstedt, 1999). It remains incompletely understood how these phenomena are generated from a molecular or mechanistic point of view. However, there is experimental evidence that the osmotic shock is perturbing the function of vesicle trafficking machineries, which thereby indirectly results in Golgi fragmentation. For instance, hypo-osmotic shock results in a reduction in the number of export sites on the ER (Lee and Linstedt, 1999). Thus, there is less trafficking from the ER to the Golgi, a condition described by others to result in alterations of Golgi structure (Cutrona et al., 2013). The identity of the signaling pathways that lead to these phenomena remains elusive. In addition, hypo-osmotic conditions seemed to also potentiate retrograde transport from the Golgi to the ER (Lee and Linstedt, 1999). Again, how this effect is mediated is totally unclear. Contrary to this, hyper-osmotic conditions seemed to only affect anterograde transport from the ER to the Golgi, but did not seem to have any major effect on retrograde transport (Lee and Linstedt, 1999). How different osmotic conditions differentially affect membrane trafficking and what the (patho) physiologic significance of his might be remains to be determined.

Recently, DNA damage induced by camptothecin, doxorubicin, or by ionizing radiation was also shown to induce dispersal of the Golgi (Farber-Katz et al., 2014). Of note, this effect was not related to the induction of apoptosis, a condition that also disrupts the Golgi. In support of the notion that this effect is a pure stress response was the elucidation of the signaling pathway that is behind this phenomenon. DNA damage was shown to activate a kinase called DNA-PK, which in turn phosphorylates the Golgi protein GOLPH3 (Farber-Katz et al., 2014). GOLPH3 is a protein that localizes to the trans-Golgi by binding to phosphatidylinositol-4-phosphate. At the Golgi, GOLPH3 mediates an interaction with the actin cytoskeleton by binding to MYO18A (Dippold et al., 2009). Phosphorylation of GOLPH3 by DNA-PK results in increased binding to MYO18A, thereby increasing the tension on Golgi membranes exerted by the actin cytoskeleton, which ultimately leads to Golgi fragmentation. At the same time trafficking from the Golgi to the cell surface is disrupted. This response of the Golgi seems to mediate a resistance of the cell to the DNA damaging agent (Farber-Katz et al., 2014). An important finding of this work was that the Golgi fragmentation phenotype was persistent and was observed to some extent for a month after the damaging agent was removed (Farber-Katz et al., 2014). This observation could be interpreted in two ways: either Golgi fragmentation is a protective (adaptive) response that persists in order to maintain cell fitness for prolonged periods of time. Alternatively, Golgi fragmentation is a pathologic response and a sign that cell fitness is reduced or that the cell is now more prone to other damaging agents.

Altogether, it appears that the Golgi apparatus is subject to extensive regulation by different classes of stressors. We are aware that it might be relevant to discriminate between different types of stress. However, for the sake of simplicity, we will only generally talk about stress and its connection to the Golgi, without going into detail of the different types of stress.

## Systematic approaches investigating regulators of the golgi

The advent of systems biology opened up new avenues to study various biological processes in a holistic manner. In the current paper we focus on RNAi screens, as these will form the basis for our computational inference strategy. To date two RNAi screens exist that aimed at identifying genes that when depleted result in a structural alteration of the Golgi (Chia et al., 2012; Millarte et al., 2015). We are aware of other screens of the secretory pathway that were recently discussed (Farhan et al., 2010; Zacharogianni et al., 2011; Simpson et al., 2012; Farhan, 2015) and we will discuss here reasons for their (partial) exclusion. Firstly, the Golgi modulating hits from the first kinome and phosphatome screen of the human secretory pathway (Farhan et al., 2010) are included in a more recent work (Millarte et al., 2015). All other hits in that screen did not affect the Golgi and therefore will not be considered for our analysis. The first screen in *Drosophila* for regulators of the secretory pathway (Zacharogianni et al., 2011) cannot be considered because the structure of the Golgi in fly cells is slightly different from mammalian cells. Indeed, the Golgi in mammalian cells is a single copy organelle that fragments in response to external or internal signals. In *Drosophila*, there are multiple pairs of Golgi stacks per cell (Kondylis et al., 2007; Kondylis and Rabouille, 2009) and therefore it is very difficult to assess the impact of the *Drosophila* screen on the mammalian Golgi apparatus. We therefore, decided not include these hits for further analysis.

We first considered the candidates identified by Chia et al. who screened the human kinome and phosphatome searching for regulators of the Golgi. This work revealed 159 Golgi-regulating genes, with several enriched sub-networks such as phospholipid metabolism or the acto-myosin cytoskeleton (Chia et al., 2012; Table 1). While the enrichment of these modules is not surprising in light of previous research, the work also revealed that several components of mitogen activated protein kinase (MAPK) pathways are potentially involved in Golgi regulation. This includes the so-called stress-activated MAPK family members, supporting the notion that the Golgi is a stress-sensing organelle. Another potential Golgi-regulating MAPK family member is ERK8 (also known as MAPK15). ERK8 was found to partially localize to the Golgi and to negatively regulate O-glycosylation (Chia et al., 2014). Notably, ERK8 was found earlier to mediate the stress response of nutrient deprivation to the ER exit sites (Zacharogianni et al., 2011). Therefore, ERK8 might be considered as a potential link between stress signaling and the Golgi.

Table 1**List of Golgi regulating proteins identified from RNAi screens**.

**Table:** 

**A**		
**Hits from Millarte et al. (2015)**		
ABL1	MGC45428	RYK
AKAP28	NCOA3	SH3RF1
BCR	NEDD9	SNARK
BLK	NYD-SP25	SRC
BMP2K	P38IP	SSH1
CASP10	PAK4	STK22B
CBL	PARD6A	STK22C
CCM2	PDCD10	STYX
CD44	PIK3C2B	TLR4
CDC42	PLCE1	TMED7
CDK4	PLCG1	TRRAP
CLTC	PLCG2	UNC119
DUSP23	PLK3	ZAP70
FYN	PRKACA	
GOLGA2	PRKCA	
HRAS	PRKCM	
KIT	PRKCZ	
LYN	PSPH	
MAPK1	PTPRC	
MAPK10	PTPRN	
MAPK14	RAC1	
MAPK8IP3	RAF1	
MAPK9	RGS2	
MAPRE1	RIPK1	
MBL2	RP6-213H19.1

**Table:** 

**B**							
**Hits from Chia et al. (2012)**							
ACPT	CHKA	EPM2A	LCP2	NPR2	PPP2R2B	ROS1	TXK
ACYP1	CKM	ERBB3	MALT1	NRBP1	PPP2R5E	RP6-213H19.1	ULK4
ADCK1	CLK1	EVI1	MAP2K7	NRG3	PPP3R1	RPRD1A	VRK3
ADCK5	COL4A3BP	EXOSC10	MAP3K13	PAG1	PRAGMIN	RPS6KB1	WNK3
AK3L1	CSNK1A1L	FBP2	MAP3K2	PAK1	PRKAG1	RPS6KB2	YSK4
AK7	CSNK1E	FGFR1	MAP3K8	PAK3	PRKAG3	SCYL3	YWHAH
ALPK2	CSNK1G1	FGFR2	MAP4K2	PANK3	PRKCE	SHPK	
ALPP	CSNK1G2	FLT3	MAP4K3	PAPSS1	PRKCSH	SPHK1	
ANGPT4	CSNK2B	GALK1	MAPK11	PAPSS2	PRKX	SQSTM1	
ANP32E	CXCL10	GAP43	MAPK15	PDGFRA	PRPS1L1	SRMS	
AURKB	DCLK2	GTF2H1	MAPKAPK5	PDK4	PTEN	SRPK2	
AXL	DGKD	HCK	MAPKSP1	PFKP	PTK2	STK32A	
BCKDK	DGKQ	HIPK1	MARK4	PHKG1	PTK7	STK36	
BMPR1B	DGKZ	HIPK3	MAST1	PHKG2	PTP4A1	STK4	
BMX	DLG3	HIPK4	MGC16169	PI4KA	PTP4A3	STK40	
BUB1	DMPK	HK1	MGC42105	PI4KB	PTPN14	TAOK2	
CAMK1	DUSP2	IGBP1	MKNK1	PINK1	PTPRA	TESK1	
CCRK	DUSP22	IGF1R	MKNK2	PIP5K1A	PTPRD	TGFBR1	
CDC2	DUSP6	IKBKE	MPP2	PKLR	PTPRF	TNIK	
CDC25A	DUSP8	INPP1	MTMR1	PPM1D	PTPRN2	TNS3	
CDC2L2	EIF2AK2	IPMK	MYO3B	PPM1F	PTPRT	TRIB1	
CDC42BPA	ENPP7	ITK	NEK11	PPM1L	PTPRU	TRPM7	
CDC42BPG	EPHA1	ITPKA	NEK2	PPP1R11	PXK	TSKS	
CDKL2	EPHA8	ITPKB	NLK	PPP1R2P9	RIPK2	TTK	
CDKN1B	EPHB1	KHK	NME2	PPP2CA	ROCK1	TWF2

**Table:** 

**C**		
**Hits from Simpson et al. (****2012****)**		
ABHD1	GPT	RADIL
ABHD5	GRIN2C	RHOU
AC068775.1	GSG2	RIOK3
ACTR3	IL18R1	RXRA
AKAP5	INADL	SIRT2
AR	INPP5J	SOS2
ARHGAP12	KIF1C	SPRYD4
ARHGAP32	KIF26A	SRSF1
ARHGAP44	KIFAP3	SST
B3GAT2	KIRREL	STC1
C1orf201	KRT6B	SYT1
C1S	LRP4	SYT7
C22orf45	LUC7L3	TALDO1
CCDC124	LVRN	TANC2
CCR4	MAGIX	TM4SF19
CKAP2	MAPK15	TMTC1
CLPB	MAST3	TRIM41
CLSTN3	MXD4	TSPAN1
COPB2	NT5C	TTC37
COPG	NUDT4	TYRO3
CTGF	PLEKHM2	UBE2E1
DENND4C	PML	WDR75
EPDR1	PPFIA1	YY2
FAM177B	PROC	ZNF503
GLRB	PRR4	ZNF512
GPBP1	PTBP1	ZNF830

Second, we have recently screened a focused library of 103 genes for effects on Golgi structure and identified at least 70 potential regulators of Golgi structure (Millarte et al., 2015; Table 1). The goal of that screen was to identify new regulators of cell migration, but it nevertheless serves (together with the Chia screen) as a repository for proteins that affect the Golgi, either directly or indirectly.

A third screen from which we partially derive hits for our computational analysis is from the first full-genome screen in human cells for regulators of secretion (Simpson et al., 2012). While this screen identified over 600 hits that regulate the secretion of a transmembrane cargo protein, we largely lack information on the impact of these hits on Golgi morphology. However, we found 78 hits that were tested for their impact on Golgi structure (Table 1) and we will include these into our collection of hits that affect Golgi morphology. All these Golgi modulating hits from the three RNA screens will be used to assemble a network of Golgi regulators, which we will link computationally to genes known to be involved in stress signaling.

## Hypothesis

**Figure 1:**
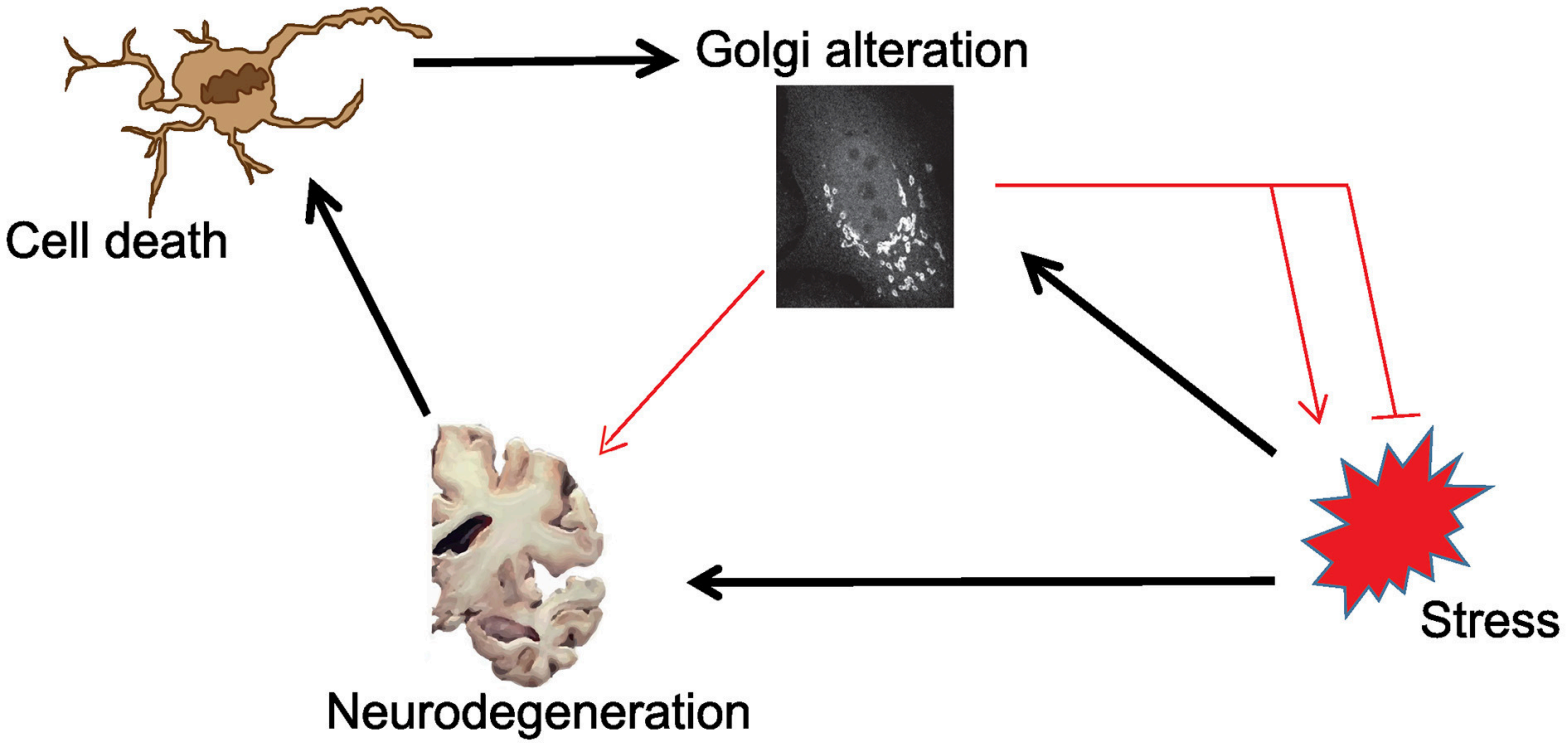
**Schematic illustration of the possible connections in the tripartite link between stress, Golgi alterations and neurodegeneration**. Black arrows indicate links that are sufficiently supported by evidence from the literature. Red connections are those that are currently of unknown relevance.

As outlined in the introductory section, it is not clear whether the stress-Golgi connection is relevant for the pathogenesis of neurodegenerative disorders. In Figure 1, we schematically illustrate this relationship and the known (black arrows) and unknown connections (red arrows). As can be seen, it is unclear whether and how Golgi alteration contributes to neurodegeneration. What is also unknown is whether Golgi alteration mends or deteriorates stress signaling. Based on this illustration the following hypothetical possibilities exist:

**Scenario 1:** Golgi fragmentation is only a byproduct of neuronal cell death and has no role in the pathogenesis of ND.
**Scenario 2:** The Golgi is altered consequently to stress signaling, but this is only an epiphenomenon that has no further role in the disease.
**Scenario 3:** The Golgi fragments in response to stress as a response of the cell to fight the stress. Here Golgi fragmentation is a protective event and interfering with this response is not a desired therapeutic option.
**Scenario 4:** Stress signals results in Golgi fragmentation, which acts as a positive feedback module that enhances stress signaling and enhances neurodegeneration. Here, blocking Golgi fragmentation is a desired therapeutic strategy.

In scenarios where Golgi alteration is not linked to the disease (scenarios 1, 2), we expect that while Golgi and stress regulators might form a network, this network will be largely devoid of processes of any relevance to neurodegeneration. In the other scenarios the Golgi is on one hand a receiver of signals (in this case stress signals) and then plays either a protective (scenarios 3) or damaging (scenario 4) role. Our approach is compatible with the Golgi being a receiver of signals in the first line, because the computational inference will be based on hits that were identified as potential regulators of Golgi organization. Thus, the Golgi can be considered a received of signals transduced by these regulators. As for the possibilities that the Golgi sends signals, we would like to emphasize that our approach cannot easily distinguish this possibility from others. Nevertheless, as will been discussed below, we inferred few hypothetical cases where the Golgi might respond to stress signaling, to in turn emit signals that contributes to neurodegeneration.

## Computational inference of a network linking stress pathways to regulators of golgi morphology

PPI orchestrate a wide range of biological process and studying PPI networks has become increasingly important and valuable in solving biological questions. Based on the three RNAi screens discussed above (Section The Golgi in Neurodegenerative Diseases), we have a collection of 320 hits (Table 1) that affect the morphology of the Golgi. In addition, we performed a search of the literature for proteins that were known to be involved in the response to the following types of stress: Osmotic stress, oxidative stress, ER stress (and unfolded protein response), radiation stress, inflammation stress, and DNA damage response producing a list of 76 proteins (Table 2). This list includes canonical components of stress signaling pathways. We are aware of studies that analyzed changes of the proteome in neurons that respond to stress (Herrmann et al., 2013). However, the list of up- and down-regulated proteins represents the end-product of stress signaling, rather than components of stress pathways. Our aim is to investigate stress signaling at the Golgi and therefore, we decided to focus on components of stress signaling pathways.

**Table 2:** **Stress-related proteins**.

DAXX	FADD	MAPK8
ALKBH8	Fas	MAPK9
ALS2	FXYD2	MAPKAPK2
APOE	GADD45A	MAPKAPK3
ATF4	GPX1	MAPT
ATF6	GPX3	MEF2C
ATM	GPX6	NEFH
BAD	GSK3B	NEFL
BCL2	HSPB1	NEFM
BID	HSPB2	NFATC1
CACNA1A	JUN	NFATC3
CAPN1	JUND	NFE2L2
CAPN2	KEAP1	PAK1
CAPNS1	LPL	PPM1B
CASP12	MAP2K3	PPP2CA
CDC25B	MAP2K4	PRKDC
CDK5	MAP2K6	PRPH
CDK5R1	MAP2K7	RELA
CSNK1D	MAP3K1	SOD1
CSNK2A1	MAP3K11	TRAF2
DDIT3	MAP3K5	TXN
DUSP1	MAP3K7	TXNRD1
DUSP4	MAPK10	ZAK
EEF2K	MAPK11	
ERN1	MAPK12	
ERN2	MAPK13	
	MAPK14	

After removing overlapping proteins between the list of stress and Golgi hits, we obtained an input list containing a total of 389 proteins that form the basis of the network inference. In this paper, the background interactome was built upon the BIOGRID database, which only relies on interactions that are supported by experimental evidence, and it is comprised of 6516 proteins and 174411 interactions. The source file can be obtained freely without the need for a license. In order to infer the network connecting the 389 proteins we looked for the connected subnetworks with the minimum number of proteins required to link them. This search was performed by solving, via mathematical programming techniques, the so-called Steiner Tree problem.

The Steiner Tree problem is a classical problem in the field of Combinatorial Optimization (Magnanti and Wolsey, 1995). A network (in our case the entire human interactome) can be defined as N = N(V,E). V are the nodes (all the human proteins) including a subset of so-called terminal nodes T (that is, the input nodes, i.e., Golgi hits and stress genes). E are the interactions between the nodes (the edges linking the nodes). The Steiner Tree problem finds a sub-network connecting all the elements in T (input nodes) using the minimum possible number of edges. Achieving such connection might require the use of additional nodes that were not originally among the input nodes. These additional nodes are called “Steiner nodes” and in this context they can be regarded as proteins that enable a functional linkage among the proteins comprised in T.

**Figure 2:**
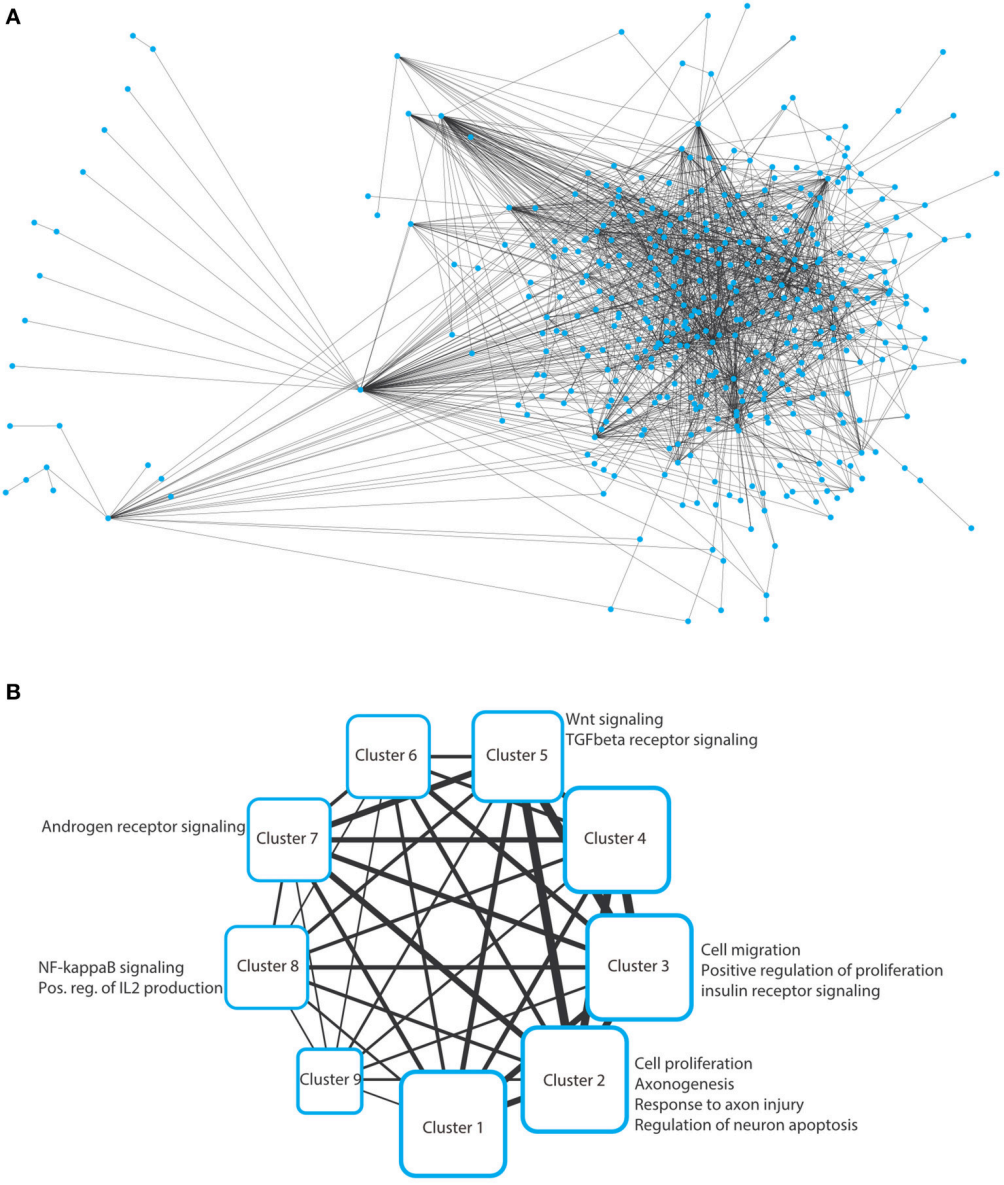
**(A)** Hairball representation of the network of protein–protein interactions between the Golgi and the stress-related proteins. The network was visualized and arranged using Cytoscape (organic style). The list of the interactions in this network are provided in Supplementary Table S1. **(B)** Nine clusters were obtained by analyzing the network in panel a using ClustNsee. Next to each cluster, we provide the most notable cellular processes that were obtained using a Gene Ontology analysis using the BinGO plugin of Cytoscape. These cellular processes are discussed in detail in the main text.

To solve this optimization problem, we use mathematical programming techniques; in particular, a specially tailored algorithm able to solve the Steiner Tree problem on the considered interactome in few seconds (Fischetti et al., 2014). Note that if it is not possible to connect all the elements in T, then the algorithms seeks for a sub-network spanning as many elements in T as possible, using as few edges as possible. The network resulting from this inference is depicted in Figure 2A as a typical “hairball” diagram to illustrate the strong connectivity in the network. Of the 389 input nodes, only 28 nodes do not appear in the network due to poor connectivity (proteins that have no known interaction partners). In addition, 57 additional nodes (Steiner nodes) were included to allow formation of a fully connected network, which indicates that stress pathway components and putative Golgi regulators are reasonably well connected.

We next asked which cellular processes are enriched in this network. This can be performed using a functional annotation clustering of gene ontology (GO) terms. However, we decided not to perform this analysis on the large network, because we will not be able to easily determine to which subnetworks a given enriched cellular process belongs. Therefore, we first aimed at de-bulking the large network and search whether it contains sub-networks. We used the ClustNsee plugin in Cytoscape to search for clusters (Spinelli et al., 2013). The accuracy of the clustering algorithm was verified using a test-network containing very closely related proteins regulating COPI vesicle formation, COPII vesicles formation, and nuclear pore components. ClustNsee could faithfully distinguish subnetworks containing the three aforementioned cellular processes (data not shown). Using the clustering algorithm on our network shown in Figure 2A revealed the presence of nine clusters, which revealed several processes and signaling pathways of relevance to neurodegeneration (Figure 2B). Next we will discuss these nine clusters.

## Relevance of the inferred sub-networks for neurodegeneration

We analyzed the different clusters using the BinGO plugin into Cytoscape. BinGO determines the statistical enrichment of GO term annotations in biologic networks. We set the threshold to a statistical significance of ≤ 0.01 after Bonferroni correction. Clusters 1, 4, 6, and 9 did not reveal any notable enrichments of cellular processes and will therefore not be discussed further.

### Cluster-2

#### CDK5

The most notable processes is the enrichment of the GO term “regulation of neuron apoptosis” (Figure 2) which included the proteins CDK5, CDK5R1, GPX1, and BCL2. As has been outlined above (Section The Golgi in Neurodegenerative Diseases), CDK5 is a serine/threonine kinase that was implicated by several groups to play a role in neurodegeneration (Liu et al., 2015). With respect to the Golgi, CDK5 was shown to phosphorylate the Golgi matrix protein GM130 and to thereby cause fragmentation of this organelle in a model of AD (Sun et al., 2008). CDK5 is also activated in response to neuronal stress and was shown to result in a defect in axonal transport, which is essential for neuronal viability (Klinman and Holzbaur, 2015).

**Figure 3:**
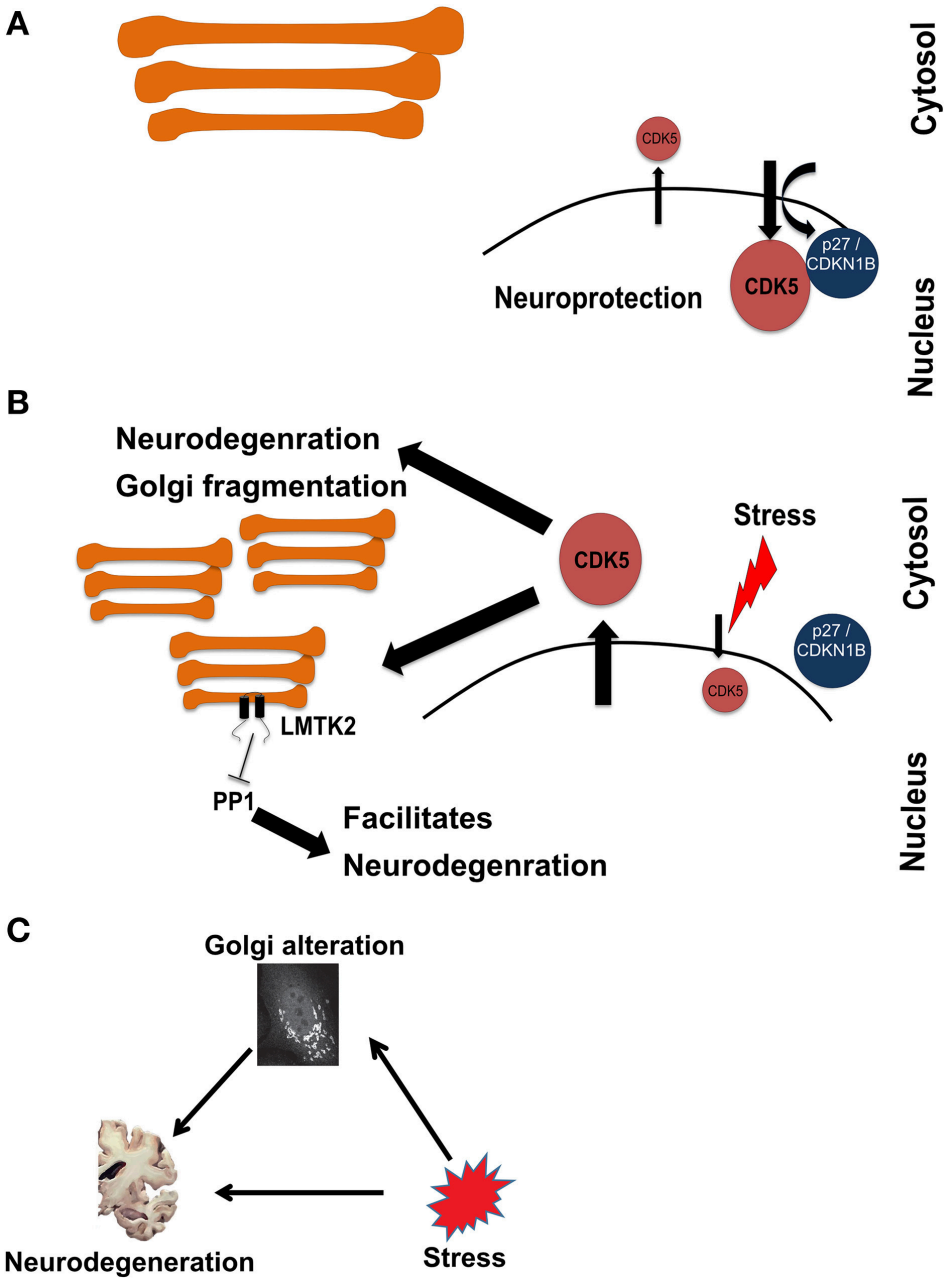
**Schematic representation of a hypothetical pathway in neurodegeneration involving stress signaling to and from the Golgi**. **(A)** Under homeostatic conditions, CDK5 is mainly kept in the nucleus in a manner dependent on p27. **(B)** Upon induction of stress, CDK5 accumulates in the cytosol, which is an event that promotes neurodegeneration. At the same time CDK5 signals to fragment the Golgi and activate LMTK2 at this organelle. LMTK2 then in turn signals from the Golgi to inhibit PP1, an event that also promotes neurodegeneration. **(C)** Schematic of the hypothetical connections between stress, Golgi alteration, and neurodegeneration, drawn in analogy to the schematic in Figure 1.

Therefore, the cumulative evidence suggests that stress-activated CDK5 is involved in inducing neurodegeneration as well as Golgi fragmentation. We therefore looked into the local network of CDK5 within this cluster to search for more potential connections. The local interactome of CDK5 includes several other cyclin-dependent kinases, which are well-known to regulate the cell cycle. This explains why cell proliferation was one of the processes that were enriched in Cluster-2. However, neurons are post-mitotic and the presence of CDK5 in the nucleus was shown to play a role in suppressing the cell cycle in neurons (Zhang et al., 2010). The nuclear import of CDK5 is dependent on p27 (labeled as CDKN2B in the network in Figure 3). Inhibition of nuclear localization of CDK5 and its concomitant accumulation in the cytosol is considered an event that triggers neuronal cell death. Treatment of neurons with beta-amyloid, a major pathogenic protein in AD, was shown to disrupt the CDK5-p27 interaction and to result in cytoplasmic accumulation of CDK5 (Zhang et al., 2010). Thus, our approach captured this connection between stress, neurodegeneration and Golgi fragmentation and is therefore an indication that our approach is valid to uncover further evidence for this tripartite connection.

The next question that we asked above is whether the stress-induced Golgi fragmentation is part of the pathologic process. In other words, is the Golgi that receives stress signaling, in turn sending signals that contribute to neurodegeneration? We therefore searched the literature for kinases with known Golgi localization that would be activated by CDK5. Intriguingly, we identified LMTK2 (also known as CPRK) as such a candidate. LMTK2 is kinase with two transmembrane domains that localizes to various intracellular membranes, including the Golgi apparatus and is known to be a target for CDK5 (Kesavapany et al., 2003). LMTK2 is known to bind to and inhibit the protein phosphatase PP1 (Wang and Brautigan, 2002; Manser et al., 2012). Inhibition of PP1 is long considered a contributing factor in the pathogenesis of AD (da Cruz e Silva et al., 2004; Liu et al., 2005). We hypothesize that this is a case where stress signals to the Golgi. This leads to structural and functional changes of this organelle that contribute the neurodegeneration (Figure 3). In the current case, stress activates CDK5, which phosphorylates the neurofilament protein tau, an event of relevance to AD (Stoothoff and Johnson, 2005). Concomitantly, stress signaling also targets the Golgi, leading to its fragmentation (Sun et al., 2008) and to activation of a protein at the Golgi (LMTK2) that itself contributes to neurodegeneration. Whether this hypothesis is true requires experimental testing. In addition, whether the fragmentation of the Golgi is itself a contributing factor also remains unclear.

#### APOE

Other processes of interest to neurodegeneration were axonogenesis and the response to axon injury. One of the proteins that plays a role in these processes is APOE, a stress-relevant protein that plays a role in axonogenesis. The human genome encodes for three alleles of ApoE: ApoE2, ApoE3, and ApoE4. The latter allele was linked to AD (Hyman et al., 1996). ApoE4 overexpression in mice results in axonal degeneration (Tesseur et al., 2000). Particles containing Apolipoprotein E (LpE) are produced by astrocytes in the brain and play a crucial role in lipid metabolism in this organ. Apoptosis in response to starvation-stress was prevented by LpE in a manner dependent on a PLCG1-PKC signaling axis (Hayashi et al., 2009). Of note, we have recently identified PLCG1 as a regulator of secretory trafficking and of Golgi structure (Millarte et al., 2015). Therefore, it might be that ApoE-to-PLCG1 signaling is involved in the modulation of neuronal fitness and of stress response. Whether this is the case remains to be tested in the future.

Among the Golgi hits, APOE is connected to TYRO3 and to MAST1. TYRO3 is a transmembrane tyrosine kinase, with unknown relevance for stress signaling. However, TYRO3 overexpression was found to counteract the production of beta-amyloid production and its deletion in mice increases the number of amyloid plaques (Zheng et al., 2012). MAST1 is a microtubule-associated kinase, that is poorly investigated and so far no clear connection between MAST1 and neurodegeneration or stress exists.

### Cluster-3: small GTPases and PAK

**Figure 4:**
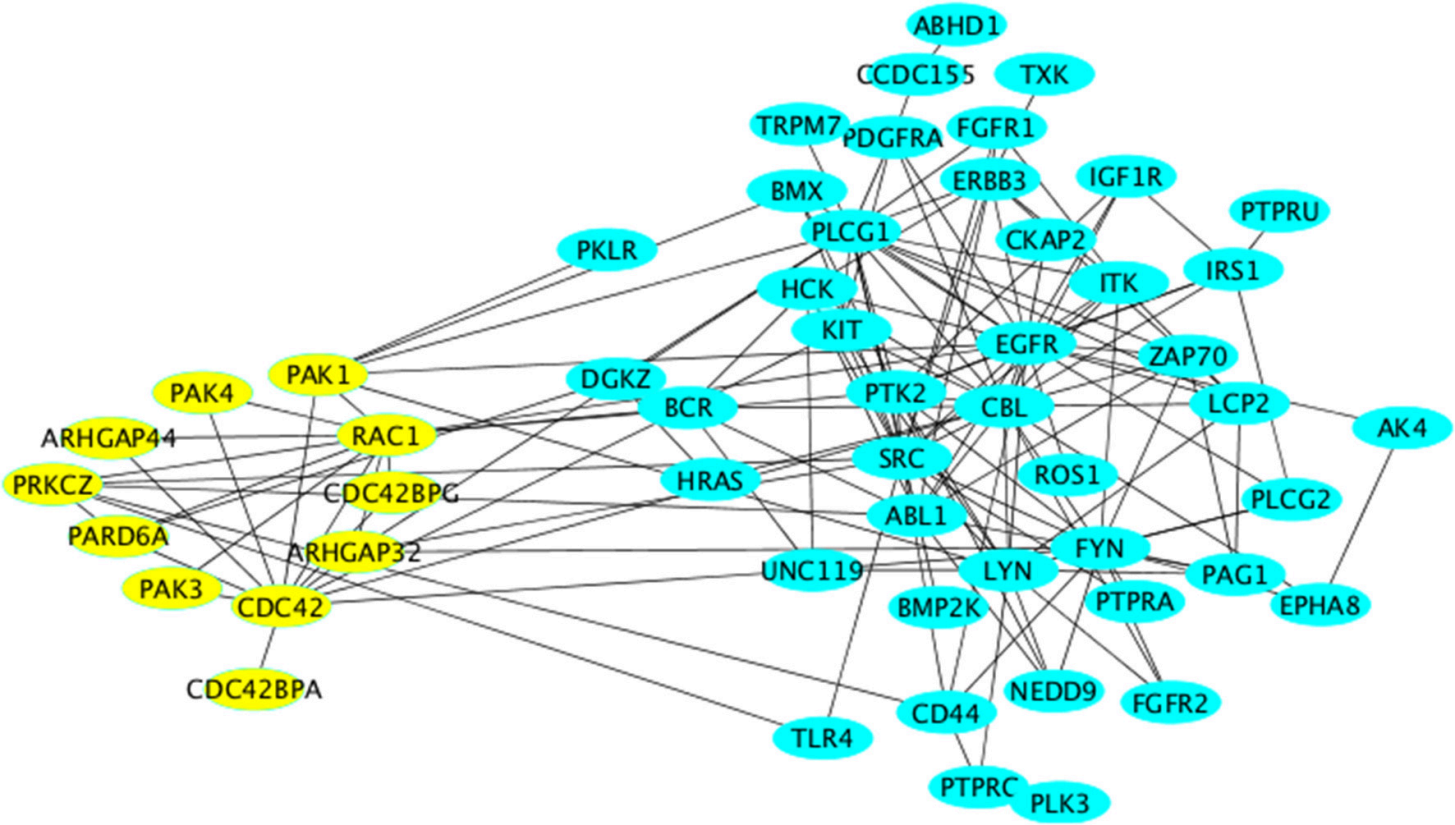
**Network representation of Cluster-3 using the “organic” style in Cytoscape**. All nodes relevant to Rho GTPase signaling are labeled in yellow and are arranged using the “circular” style in Cytoscape.

In this cluster, we again obtained processes relevant to proliferation as well as mitogenic signaling (insulin signaling). The main proteins within these processes were components of classical growth factor cascades (HRas, receptor tyrosine kinases, etc…). The enrichment of these processes is probably best explained by the fact that these pathways are survival pathways and therefore act to counteract neuronal death. Another interesting biologic process “cell projection organization,” which goes in line with the enrichment of axonogenesis in Cluster-2. However, the enrichment of this process here was mainly due to the presence of the cytoskeletal regulator Rac1, which belongs to the Rho family of small GTPases. We noted the presence of other members, regulators and effectors of Rho GTPase signaling were also found here (yellow nodes in Figure 4).

We searched for evidence for a role of Rho GTPase signaling in neurodegenerative diseases. The PAK kinases are activated downstream of the two Rho family GTPases Cdc42 and Rac1 (both present in Cluster-3). The two major neuronal isoforms are PAK1 and PAK3, which are both found in the subnetwork depicted in Figure 3B. Mutations of PAK3 were shown to causally underlie nonsyndromic X-linked mental retardation (Allen et al., 1998). PAKs also mediate the pro-survival effect of the Rho family GTPase Rac1 (Johnson and D'Mello, 2005; Loucks et al., 2006). Although the cognitive dysfunction in AD patients correlates well with the formation of amyloid plaques and neurofibrillary tangles, there is a subset of patients who have only little (or no) signs of such amyloid formation. In general the correlation between the histopathological signs of the disease and the clinical symptoms is far from being linear (Nelson et al., 2009). Therefore, it is likely that another factor contributes to the disease in addition to neuronal cell death. One such factor might be the alteration of neuronal function such as the formation of synapses. Based on our network analysis we suggest that stress signaling affects signaling by Rho GTPases and by PAKs leading to alterations of synaptogenesis, which might manifest itself as cognitive defects. In support of this is the observation that DOCK3, an activator for Rac1 exerts a cyto-protective effect on neuronal cells exposed to oxidative stress (Namekata et al., 2013, 2014). Furthermore, Huntingtin-induced neurotoxicity was modulated by Rac1 and PAK2, thus supporting our suggestion that the role of Rho GTPases and PAKs in neurodegeneration should be reexamined in the context of stress signaling.

ALS is a neurodegenerative disease thought to involve inflammation and oxidative stress. The ALS-causing mutant protein Cu(+)/Zn(+) superoxide dismutase SOD1-G93A directly enhances the activity of the main ROS-producing enzyme in microglia, NADPH oxidase 2 (NOX2; Bedard and Krause, 2007). A major activator of NOX2 is the Rho-GTPase family member Rac1 (Hordijk, 2006), implying that Rac1 might be involved in neurodegeneration. Rac1 was also shown to be involved in mediating the neurotoxic effect of 1–42 β-amyloid peptides (Manterola et al., 2013). A similar effect of these amyloidogenic peptides was observed toward Cdc42, an effect that was further shown to affect the actin cytoskeleton (Mendoza-Naranjo et al., 2012). Notably, the GTPase Cdc42 was not only shown to localize to the Golgi, but also to be regulated at this compartment (Baschieri et al., 2014). It is unclear whether stress signaling affects Cdc42 at the Golgi and if so, whether this is of any relevance to neurodegeneration. At the moment, our network inference can only hypothesize about the existence of such a connection and certainly future research on endomembrane signaling of Rho GTPases will reveal more insights.

### Cluster-5: Wnt and CK?

From the BinGO analysis of this cluster, we noted the enrichment of Wnt signaling and transforming growth receptor beta (TGF-beta) signaling (Figure 2B). It was proposed previously that Wnt signaling might play a protective role in neurodegeneration (for a review see Arrázola et al., 2015). However, our aim was to search for evidence for the relevance of the connection between Golgi, stress, and neurodegeneration. The enriched proteins within Wnt signaling where two members of the casein kinase 1 (CK1) family (CSNK1G1 and CSNK1G2), which were hits that were found to regulate Golgi structure (Chia et al., 2012). CK1 signaling is in general considered to inhibit Wnt signaling. Together with the notion that Wnt signaling is neuroprotective, it might be that activation of CK1 by stress could facilitate neurodegeneration via inhibition of Wnt signaling. With respect to the Golgi, members of the CK1 family were shown to be important for maintaining Golgi integrity and for trafficking to the Golgi (Greer et al., 2014). Cells stressed by misfolded proteins were shown to exhibit CK1-dependent phosphorylation of parkin (Yamamoto et al., 2005), a protein mutated in Parkinson's disease. Whether alzerations of Golgi structure modulate CK1 activity, or whether the modulation of CK1 activity itself has an impact on the Golgi in neurodegeneration cannot be addressed at the moment. Nevertheless, our network inference should motivate toward research on the role of CK1 signaling and its modulation of endomembrane traffic in neurodegeneration.

TGF-beta1 is known to exert a neuroprotective effect and to counteract neurodegenerative disease such as AD (Caraci et al., 2012). However, what is the connection to the Golgi biology and stress signaling? It was shown that a brief ischemic stress of the brain results in a stronger colocalization of TGFbeta1 with Golgi membranes, indicating a defect in its secretory trafficking out of the Golgi (Hu et al., 2007). Concomitantly, the study also reported aberrant Golgi morphologies. Therefore, hypoxic stress of in neurons might result in alterations in trafficking of TGF-beta and are therefore expected to result in a reduction in TGF-beta signaling and a loss of its neuroprotective effects. In this scenario, alterations of the Golgi are an active part of the disease process. Whether this mechanism is true, or whether it is relevant for other stresses remains to be elucidated in the future.

### Cluster-7: the androgen receptor signaling pathway

In this cluster, we noted the enrichment of “androgen receptor signaling pathway,” prompting us to ask whether androgens play a role in the connection between stress, neurodegeneration, and the Golgi. Androgens are known to play a role in the pathogenesis of different neurodegenerative disorders. Disruption of androgen signaling was suggested to play a role in the pathogenesis of ALS and spinal bulbar muscular atrophy (SBMA), because the expression of the androgen receptor was found to be downregulated in motoneurons of diseased mice (Sheean et al., 2015). Because women are at twice as high risk to develop AD (Viña and Lloret, 2010), it has been speculated whether androgens might not represent a protective factor against this disorder. The exact reason for this sex-difference is unclear. However, the androgen receptor is located on the X chromosome, and an interesting hypothesis would be to test whether the risk for Alzheimer's correlates with the X-inactivation patterns (Ferrari et al., 2013). The androgen receptor was a hit in the full-genome screen for regulators of secretion (Simpson et al., 2012).

Nevertheless, a link to the Golgi is not self-evident. We therefore inspected the network around the androgen receptor in Figure 2A and noted the tyrosine kinase Src among the interaction partners. Members of the Src family were not only shown to reside at the Golgi, but their activation was also shown to result in Golgi fragmentation (Weller et al., 2010). Therefore, stress-induced Src signaling might result in Golgi fragmentation in neurodegenerative disorders. However, what is the biologic consequence of this Src-induced Golgi fragmentation? With respect to trafficking, Src signaling was recently suggested to activate the small GTPase Cdc42 leading to a facilitation of anterograde transport within the Golgi (Park et al., 2015). This is in line with a study that showed that Golgi fragmentation in neurons exposed to beta-amyloid facilitates secretion of amyloid precursors and thereby increases the deposition of toxic protein species outside cells (Joshi et al., 2014). Altogether, we postulate the following hypothesis: the lower levels of androgens in women and the decline of androgen levels in elder men results in reduced signaling by androgen receptor. Whether and how this modulates Src signaling at the Golgi, in particular in the presence of cellular stress requires experimental testing. We suggest that Src signaling is increased, resulting in Golgi fragmentation, which in turn facilitates the progression of the disease.

### Cluster-8: NF-kB

Tis cluster revealed the enrichment of NF-kB signaling as well as the positive regulation of interleukin-2 (IL2) production. Although interesting, the enrichment of these pathways is not surprising. These are two well-established pathways downstream of inflammatory stimuli and the notion that neuroinflammation contributes to neurodegenerative disease is well documented (see for instance the very recent review by Steardo et al., 2015). Therefore, we will not discuss this in any further detail.

## Concluding remark

Our computational inference of a network between stress regulators and RNAi screening hits identified as putative Golgi regulators has revealed a clear enrichment of processes relevant for neurodegeneration. We emphasize, that our work is theoretical in nature and cannot replace the validation using “wet lab” experiments. Further, we stress that we are well aware of the limitation that are imposed by using this list of Golgi regulators, because they were identified in cells that were cultured under optimal growth conditions and therefore we might be missing some potential stress-specific regulators. In addition, the screens were performed in HeLa cells and not in neurons. The Golgi in neurons is different from epithelial cells as it was shown to form so called Golgi outposts far away from the somatic Golgi (Ehlers, 2007). Nevertheless, the fundamental organization of the Golgi is similar as every Golgi is organized as a stack of flattened cisternae and so far the Golgi is always involved in protein sorting and post-translational modification. Therefore, insights obtained from screens in HeLa cells are of potential relevance for the functional organization in neurons. In addition, the stress pathways operating in neurons and other cell types are essentially the same. Therefore, despite these limitations we think that our work will provide a starting point for future investigation on the role tripartite connection between the Golgi, stress signaling and neurodegeneration.

Our analysis is supportive of a tripartite link between stress, Golgi alteration, and neurodegeneration. In some instances we found hypothetical evidence for a Golgi not only being a receiver of stress signals, but also that stress signaling to the Golgi triggers another signaling event that might deteriorate neurodegeneration (see scenario in Figure 3). Another is the de-regulated signaling by Rho family GTPases at the Golgi and the potential consequences for the pathogenesis of neurodegeneration (see Cluster-7 and Cluster-3). These, and other predictions we made should require experimental testing by the research community.

### Conflict of interest statement

The authors declare that the research was conducted in the absence of any commercial or financial relationships that could be construed as a potential conflict of interest.
